# Experimental benchmarking of in silico annotations at prostate cancer risk loci and hypothesis-generating cross-cancer prioritisation of ovarian risk variants

**DOI:** 10.1186/s44342-026-00087-2

**Published:** 2026-10-28

**Authors:** Wiaam Ahmed Al-Amili, Mohammed Ayyed Najm, Shatha Saadi Jumaah, Ahmed AbdulJabbar Suleiman

**Affiliations:** 1 Department of Biology, Faculty of Science, Al-Esraa University, Baghdad, Iraq; 2 Department of Pharmacology, College of Medicine, Ibn Sina University of Medical and Pharmaceutical Sciences, Baghdad, Iraq; 3 Department of Biology, Education Faculty, University of Al-Hamdaniya, Mosul, Iraq; 4 Department of Biotechnology, College of Science, University of Anbar, Anbar, Iraq

**Keywords:** Non-coding variants, Massively parallel reporter assay, Expression quantitative trait loci, Functional genomics, Pleiotropic risk loci

## Abstract

Genome-wide association studies (GWAS) have identified numerous non-coding risk variants for prostate and ovarian cancers, yet determining which are regulatorily active and which target genes they control remains challenging. We re-analysed a dinucleotide-barcode massively parallel reporter assay (DiR-seq) of 208 bi-allelic prostate cancer risk single-nucleotide polymorphisms (SNPs) in three prostate cancer cell lines under false-discovery-rate (FDR) control, identifying 50 regulatory SNPs at FDR < 0.10 (30 at FDR < 0.05), including 9 shared by at least two cell lines. Benchmarking standard in-silico functional annotations against this experimental ground truth revealed poor predictive performance (cross-validated ROC-AUC = 0.55–0.59), demonstrating that computational predictions cannot reliably substitute for measured allelic activity. We therefore treated the ovarian analysis as hypothesis-generating. Convergent annotation evidence prioritised 44 of 176 ovarian cancer GWAS leads (31 independent loci); a core of 21 SNPs (17 loci) was stable under alternative weights, leave-one-feature-out scoring and threshold changes, and candidates were enriched in ovarian and fallopian-tube regulatory chromatin (OR = 7.9, FDR = 5 × 10⁻5). Copy-number recurrence at candidate genes agreed between two tumour cohorts (Spearman ρ = 0.75, 95% CI 0.46–0.88) but no more than for chromosome-arm-matched control genes, so this observation is exploratory. Formal colocalisation with full summary statistics supported a shared causal variant for prostate cancer risk and TLCD3A expression at 17p13.3 (PP.H4 = 0.92), whereas the ovarian 17q21.31 signal could not be resolved to a single gene. Transcription-factor motifs disrupted in both cancers were over-represented for the interferon-regulatory factor (IRF) and STAT families (FDR = 3 × 10⁻6), a motif-level hypothesis. In an independent MPRA in primary ovarian epithelial cells, variants disrupting interferon-stimulated response elements (ISREs) were depleted, not enriched, for allele-specific activity (4.6% vs 9.2%; OR = 0.47, nominal *p* = 0.06, FDR = 0.10), so the IRF/STAT hypothesis was not supported; the same assay confirmed allele-specific activity at one prioritised ovarian candidate, rs2976384 (skew = − 0.67, FDR = 3 × 10⁻5). These results provide an audited prostate regulatory-SNP catalogue, quantify the limits of annotation-based fine-mapping, and offer testable cross-cancer hypotheses.

**Supplementary Information:**

The online version contains supplementary material available at https://doi.org/10.1186/s44342-026-00087-2.

## Introduction

Genome-wide association studies (GWAS) have mapped thousands of susceptibility loci for epithelial malignancies, reshaping our understanding of inherited cancer risk [1]. Prostate and ovarian cancers are two such hormone-driven malignancies with distinct clinical challenges but overlapping epidemiological and genetic risk architectures [2]. Prostate cancer is the most frequently diagnosed malignancy among men, with nearly 300,000 new US cases estimated in 2024, whereas ovarian cancer remains highly lethal, accounting for approximately 13,000 annual deaths [3]. Establishing the underlying molecular mechanisms is difficult because the majority of trait-associated variants reside in non-coding regions [1] that disrupt tissue-specific enhancers and transcription factor binding sites, obscuring the causal alleles and their target genes [4]. Resolving these mechanisms requires pinpointing causal variants within high-linkage-disequilibrium (LD) blocks and mapping them to the genes they regulate [5]—a challenge compounded by the fact that computational predictions alone may not capture true biological activity.

Studying prostate and ovarian cancers jointly offers an opportunity to uncover shared non-coding regulatory mechanisms driving tumourigenesis across sex-specific tissues. The two diseases overlap through shared polygenic risk factors and cross-cancer susceptibility loci that modulate hormone-related pathways [6], and specific loci—notably HNF1B—exert pleiotropic effects across both tissues [2]. Recent cross-cancer meta-analyses further suggest a broader shared genetic architecture, indicating that joint functional fine-mapping could expose common regulatory vulnerabilities that single-trait studies overlook [7]. Massively parallel reporter assays (MPRAs) are critical tools here, enabling high-throughput empirical quantification of allele-specific activity across thousands of sequences simultaneously [5]; deploying them across shared risk loci could clarify how pleiotropic non-coding variants influence susceptibility.

Previous studies deployed dinucleotide-barcode parallel reporter assays (DiR-seq) to interrogate 213 prostate cancer risk SNPs across multiple cell lines [8], but interpreted these data with uncorrected statistical thresholds, leaving headroom for rigorous error control and cross-cell-line synthesis. Meanwhile, the field increasingly relies on in-silico annotations—TF motif disruption, epigenomic overlap—to fine-map loci when assays are unavailable [9], yet these annotations are rarely benchmarked against an experimental ground truth. Their unvalidated use is a critical limitation, as computational predictions may fail to substitute for measured allelic activity. Regulatory activity also depends on chromatin context: epigenetic state shapes three-dimensional chromatin organisation [10, 11], and spatial proximity in chromatin links regulatory variants to their target genes in cancer tissues [12], features that neither sequence-based annotations nor episomal reporter assays capture.

We hypothesised that a definable subset of prostate and ovarian risk SNPs function as true regulatory variants driving distinct enhancer or promoter activities, and that the two malignancies share a transcription-factor programme acting on lineage- and survival-associated genes, potentially mediated by immune-regulatory networks [13]. To test this, we re-analysed the prostate DiR-seq dataset with strict false discovery rate (FDR) control, benchmarked in-silico annotations against the measured allelic activity, and transferred this framework to ovarian cancer GWAS loci. Because the benchmark showed annotations to be weak predictors of measured activity, we present the ovarian prioritisation and the cross-cancer synthesis as hypothesis-generating, and we tested the resulting interferon-regulatory hypothesis in an independent MPRA.

## Methods

### Data acquisition and preprocessing

Processed barcode-count tables and the SNP-to-barcode map were retrieved from GEO accession GSE165765, comprising SNP-construct assays in 22Rv1, PC-3, and LNCaP prostate cell lines (3 replicates each), plus MCF7 breast controls, blank/no-insert controls, and input plasmid DNA libraries. Six 2-column, ~ 1.05-million-row full 410 barcode-space bias libraries were excluded. The remaining 22 three-column construct libraries were parsed, matching barcodes to SNPs and alleles, and normalised for quality control using counts-per-million (CPM). An independent pan-cancer MPRA dataset (GSE183301 [14]; 5095 cancer risk SNVs across 14 primary human cell types, including primary prostate and primary ovarian epithelial cells; 10 barcodes/allele) was downloaded for validation. Ovarian tumour genomics were obtained from TCGA-OV (RNA-seq STAR/TPM, gene-level ASCAT3 copy number) via TCGAbiolinks (version 2.38.0) and GEO GSE41253 (68 epithelial ovarian carcinomas, Agilent aCGH). Versions and accession numbers of all resources are listed in Supplementary Table S11.

### Allelic activity modelling

Primary allelic analysis used MPRAnalyze (version 1.28.0) [15], fitted separately for each cell line (22Rv1, PC-3 and LNCaP); cell line was therefore not a model term. Within each cell line, the factor condition denotes the allele (two levels, allele 1 and allele 2 of each SNP), and replicate enters only through library depth factors. The exact specification was analyzeComparative(dnaDesign = ~ condition, rnaDesign = ~ condition, reducedDesign = ~ 1) followed by testLrt(): the DNA model estimates the input abundance of each allele construct, and the allelic effect is the condition coefficient of the transcription-rate (RNA) model, tested by a likelihood-ratio test (LRT) against the intercept-only reduced model (one degree of freedom). Replicate library depth was captured via estimateDepthFactors(lib.factor = “batch”, depth.estimator = “uq”) rather than as a design term, because the DNA libraries had two replicates and the RNA libraries three. A generic plasmid served as the DNA denominator for 22Rv1 and PC-3, and a LNCaP-specific plasmid for LNCaP. GSE165765 provides two input-plasmid sequencing libraries of the same 425-barcode SNP construct pool: a generic plasmid library (two replicates) and a separately prepared and sequenced library labelled “plasmid(LNCaP)” (two replicates), which accompanies the LNCaP experiment; each RNA library was normalised to its matched input. The two inputs were nearly identical (Pearson r = 0.998 for mean log2 CPM across the 425 SNP barcodes), and re-fitting LNCaP with the generic plasmid gave concordant allelic effects (r = 0.98) and retained all four LNCaP calls. The three tri-allelic SNPs (rs7127900, rs2238776 and rs10940579) were excluded from allelic testing because the comparative model contrasts two alleles; they were retained only for quality control. A secondary cross-check used a limma (version 3.66.0) empirical-Bayes moderated linear model on log2(RNA CPM) − log2(DNA CPM). Regulatory SNPs were defined by LRT FDR < 0.10 (Benjamini–Hochberg). This threshold was chosen because the assay has a single barcode per allele and three replicates, which limits power (particularly in LNCaP), and because the catalogue is intended for hypothesis generation; because FDR < 0.10 is permissive, the full catalogue was also re-derived at FDR < 0.05 as a sensitivity analysis (Supplementary Table S5).

### In silicofunctional annotation and benchmarking

Variant coordinates (GRCh38/hg38) were retrieved with biomaRt (version 2.66.0). Allele-specific TF motif disruption was assessed with motifbreakR (version 2.24.0) [16] against HOCOMOCO v9 position weight matrices (426 human PWMs distributed with motifbreakR; method = “ic”, threshold = 1e − 4, filterp = TRUE). Regulatory features were annotated via the Ensembl VEP REST API (regulatory = 1; GRCh38, accessed July 2026), and target genes nominated using GTEx v10 single-tissue cis-eQTLs for prostate and ovary (GTEx Portal API v2). GWAS consensus risk alleles were retrieved with gwasrapidd (version 0.99.18). To benchmark annotation predictive power, elastic-net logistic regression (glmnet 5.0, α = 0.5) and random forest classifiers (ranger 0.18.0, 800–1500 trees) were trained on annotation features to predict the experimental regulatory call, evaluated by 20 × repeated 5-fold cross-validation reporting out-of-fold ROC-AUC (pROC 1.19.0.1) and PR-AUC (PRROC 1.4).

### Ovarian fine-mapping and validation

Genome-wide-significant ovarian GWAS lead SNPs (p < 5 × 10⁻8) were queried from the GWAS Catalog and scored (0–5) on convergent evidence: strong TF-motif disruption, Ensembl regulatory overlap, promoter/enhancer overlap, GTEx ovary eQTL, and ovarian lineage-TF motif disruption (one point each; the score of every lead is given in Supplementary Table S6). ENCODE ovary and fallopian-tube chromatin overlap was not part of the score and was used for a separate enrichment test. Candidates were grouped into independent loci by distance-based clumping (greedy by p-value, 500-kb window). Survival analysis in TCGA-OV used median-split Kaplan–Meier log-rank tests and univariate Cox models (survival 3.8.3) for 43 candidate genes, with Benjamini–Hochberg correction across genes. Copy-number recurrence was evaluated with TCGA-OV (gain CN ≥ 3, loss CN ≤ 1) and GSE41253 (log2 thresholds |0.3| gain/loss). Linkage disequilibrium between prostate-tested SNPs and ovarian leads was computed with the Ensembl LD REST API (1000 Genomes phase 3, EUR).

### Colocalisation and fine-mapping

Formal colocalisation used full summary statistics for both traits. GWAS summary statistics were obtained from the GWAS Catalog for prostate cancer (GCST006085, harmonised GRCh38 file with effect sizes and standard errors; N = 140,254, case fraction 0.564) [17] and ovarian cancer (GCST004415, Ovarian Cancer Association Consortium overall epithelial ovarian cancer; N = 66,450, case fraction 0.384; GRCh37 positions matched to GRCh38 by rsID) [18]. Full cis-eQTL summary statistics for every variant–gene pair were taken from the uniformly re-processed GTEx v8 release in the eQTL Catalogue (prostate QTD000306, n = 218; ovary QTD000291, *n* = 167) [19, 20]. Analyses used fixed windows around the flagship leads (chr17:400,000–850,000 for rs684232 and chr17:45,300,000–46,600,000 for rs2532395, GRCh38), restricted to variants with harmonised alleles and minor allele frequency > 0.01; no LD pruning was applied, because colocalisation requires all variants in the region. coloc.abf (coloc 5.2.3) [21] was run with the default priors p1 = 1 × 10⁻4, p2 = 1 × 10⁻4 and p12 = 1 × 10⁻5, with sensitivity analyses at p12 = 5 × 10⁻6 and 1 × 10⁻6, and colocalisation was declared at PP.H4 ≥ 0.8. Because coloc.abf assumes one causal variant per trait, we also ran coloc-SuSiE [22], fine-mapping each trait with SuSiE-RSS (susieR 0.14.2, L = 10) [23] using an LD matrix from 1000 Genomes phase 3 European samples (n = 503) [24], and we intersected GWAS 95% credible sets with GTEx v10 SuSiE eQTL credible sets, summarising overlap as a colocalisation posterior probability (CLPP, the sum over shared variants of the product of posterior inclusion probabilities). Every gene tested in GTEx v8 within each window was analysed (Supplementary Table S8). The regional enrichment test used previously (Fisher’s exact test of eQTL variants among GWAS-associated variants) measures regional overlap only and is not used as evidence of a shared causal variant.

### Independent MPRA, robustness analyses and multiple-testing correction

GSE183301 barcode counts were re-analysed per cell type: per-barcode activity was log2(RNA CPM + 1) − log2(mean plasmid CPM + 1); allelic skew was the mean alternative-minus-reference activity across 10 barcodes per allele and three replicates, tested with Welch's t-test, with Benjamini–Hochberg FDR across all SNVs. Concordance with DiR-seq was assessed for the 16 shared SNPs separately for each DiR-seq cell line, after orienting both skews to the same alternative allele (Pearson and Spearman correlation, sign test). Robustness of the ovarian prioritisation was assessed with leave-one-feature-out scoring, four alternative weightings (eQTL × 2; chromatin features × 2; motif features × 0.5; and weights restricted to the two features with non-zero elastic-net coefficients in the prostate benchmark, motif and eQTL), alternative thresholds (score ≥ 1, ≥ 3 and ≥ 4), alternative motif and chromatin definitions, 2000 random Dirichlet weightings, and an experimental criterion (allele-specific activity in GSE183301 primary ovarian epithelial cells); SNPs selected in at least 80% of the 12 deterministic schemes and 80% of random weightings were defined as the robust core. For the cross-cohort copy-number comparison we computed a bootstrap 95% confidence interval (10,000 resamples of genes), a permutation p-value (10,000 shuffles), a jackknife, and an empirical comparison with 10,000 random control sets of 22 genes matched to the candidates by chromosome arm, drawn from all 14,045 genes measured in both cohorts. Benjamini–Hochberg FDR was applied to every hypothesis test within pre-defined families (motif and chromatin enrichment tests; survival tests across candidate genes), and adjusted values are reported alongside nominal p-values (Supplementary Table S10).

## Results

### Data assembly and quality control confirm assay reproducibility

We parsed the 28 provided count tables and barcode maps into a complete barcode-by-library matrix. Six files of ~ 1.05 million rows were full 410 barcode-space bias libraries and were excluded. The finalised matrix comprised 627 barcodes across 22 libraries with zero dropout—425 SNP-construct barcodes (208 bi-allelic and 3 tri-allelic variants, 211 unique SNPs) and 202 control barcodes. Replicate consistency was high in 22Rv1 (Pearson r ≈ 0.99) and PC-3 (r ≈ 0.94), while LNCaP was noisier (r = 0.76–0.95) (Supplementary Figure S1, Supplementary Figure S2A). Read counts confirmed adequate depth across all retained libraries (Supplementary Figure S2B). Confirming parsing accuracy, we recovered the reported headline signal for rs684232: reporter activity (RNA/DNA) was 1.55 for the T allele versus 0.89 for the C allele (two-tailed t-test *p* = 7 × 10⁻4) (Supplementary Figure S2C).

### Rigorous allelic modelling identifies a multi-cell-line regulatory-SNP catalogue

**Fig. 1:**
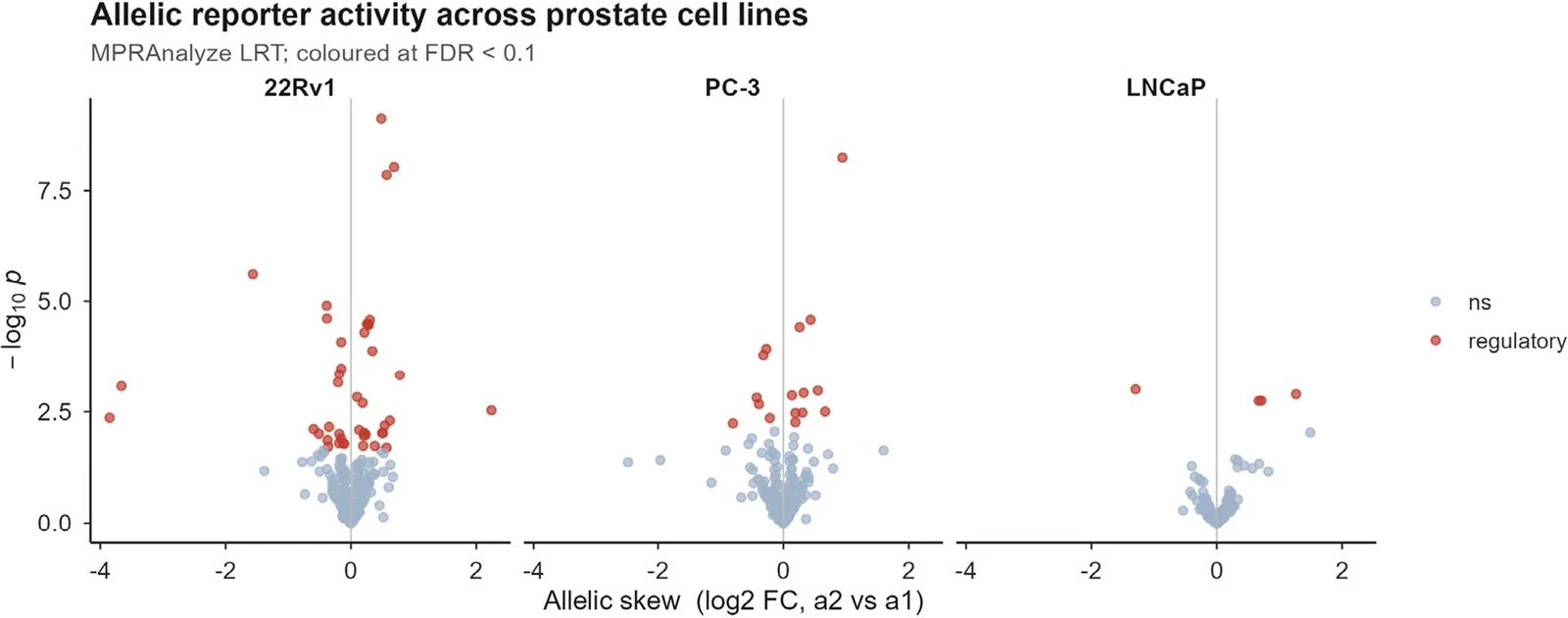
Volcano plots of allelic reporter activity across 22Rv1, PC-3, and LNCaP prostate cell lines

**Fig. 2:**
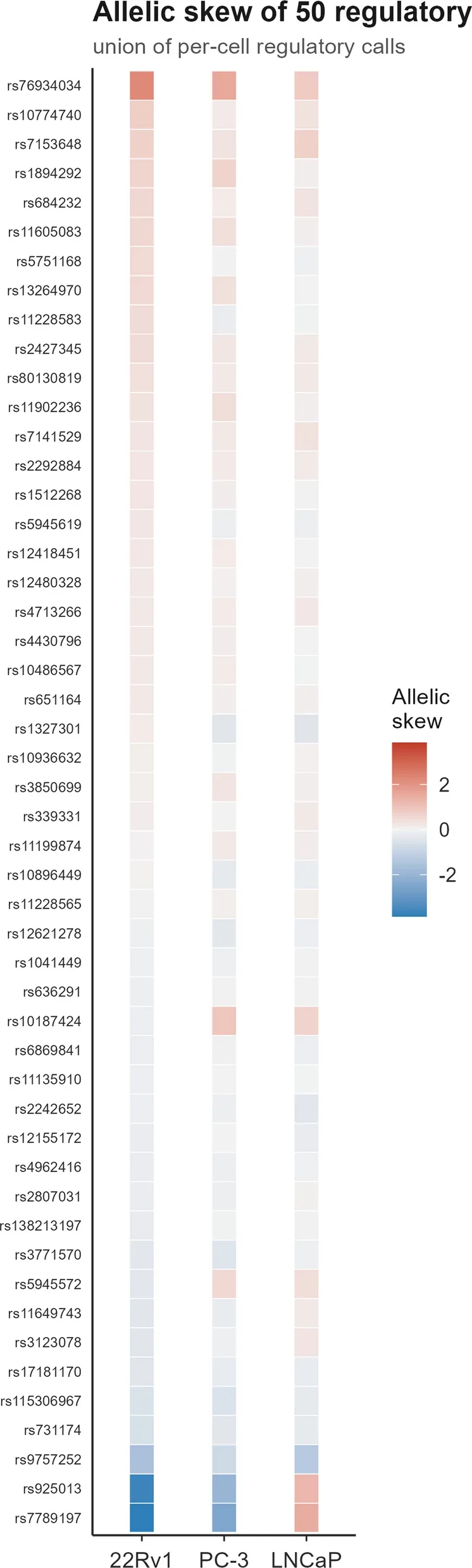
Heatmap displaying the allelic skew of the 50 identified regulatory SNPs

Replacing the original uncorrected ratio test with an FDR-controlled allelic analysis, we compared the regulatory activity of the 208 bi-allelic SNPs across the three prostate cell lines. MPRAnalyze identified 50 SNPs as significantly regulatory (FDR < 0.10) in at least one line (Supplementary Table S1, Fig. 1): 43 in 22Rv1 (23 at FDR < 0.05), 16 in PC-3 (10 at FDR < 0.05), and 4 in LNCaP (0 at FDR < 0.05). All 63 per-line calls had the same direction of effect in the limma cross-check, and 59 also reached limma FDR < 0.10. Cross-cell classification identified a robust core of 9 shared SNPs (regulatory in ≥ 2 lines with consistent direction, e.g., rs7153648, rs2427345, rs9757252), 39 cell-specific SNPs (33 unique to 22Rv1, 6 to PC-3), and 2 shared-discordant SNPs (Fig. 2, Supplementary Figure S3); the remaining 158 showed no significant activity. At the stricter threshold of FDR < 0.05, 30 SNPs remained regulatory in at least one line (27 cell-specific and 3 shared: rs7153648, rs2427345 and rs11902236; Supplementary Table S5). This set is more conservative than previous uncorrected analyses (Supplementary Figure S4A). The flagship SNP rs684232 was 22Rv1-specific (allelic log2 fold-change + 0.57 for the T allele relative to the C allele, FDR ≈ 1 × 10⁻6; that is, the C risk allele lowers reporter activity). Orienting each skew by the GWAS risk allele showed that risk alleles do not uniformly increase enhancer activity: among the 33 regulatory SNPs whose reported risk allele matched one of the tested alleles, the 22Rv1 skew was positive for 15 and negative for 18 (Supplementary Figure S4B); the other 17 regulatory SNPs had no prostate risk allele reported in the GWAS Catalog (*n* = 12) or a reported allele that did not match the tested alleles (*n* = 5), and were excluded only from risk-oriented analyses.

### In silicofunctional annotations poorly predict experimental allelic activity

Among the mapped variants (209 of 211 to GRCh38), 29 of the 50 regulatory SNPs disrupted a TF motif, 12 were prostate eQTLs, 7 overlapped an Ensembl regulatory feature, and 38 had a prostate cancer risk allele reported in the GWAS Catalog (Supplementary Figure S5). The pipeline validated against existing data: rs684232 returned significant prostate eQTLs for VPS53, TLCD3A (FAM57A), and GEMIN4, matching prior target-gene nominations.

We then trained classifiers to predict the experimental regulatory call from annotations. Annotation-only models performed poorly (cross-validated ROC-AUC 0.55 elastic-net, 0.58 random forest; PR-AUC 0.30 vs 0.24 baseline) (Supplementary Figure S6A), with only eQTL-gene count (coefficient 0.13) and motif count (0.12) receiving non-zero elastic-net weights (Supplementary Figure S6B). Adding cell-type-matched ENCODE epigenomic tracks—regulatory SNPs were enriched for open chromatin (12% vs 4%, OR = 2.9) and active marks (28% vs 18%, OR = 1.7), although neither enrichment was significant after FDR correction (FDR = 0.12 and 0.19)—only marginally changed performance (random forest AUC 0.59; elastic-net 0.53) (Supplementary Figure S6C). Thus generic and even cell-type-matched annotations are weak predictors of allele-specific MPRA activity.

### Convergent annotation evidence generates hypotheses for ovarian risk loci

**Fig. 3:**
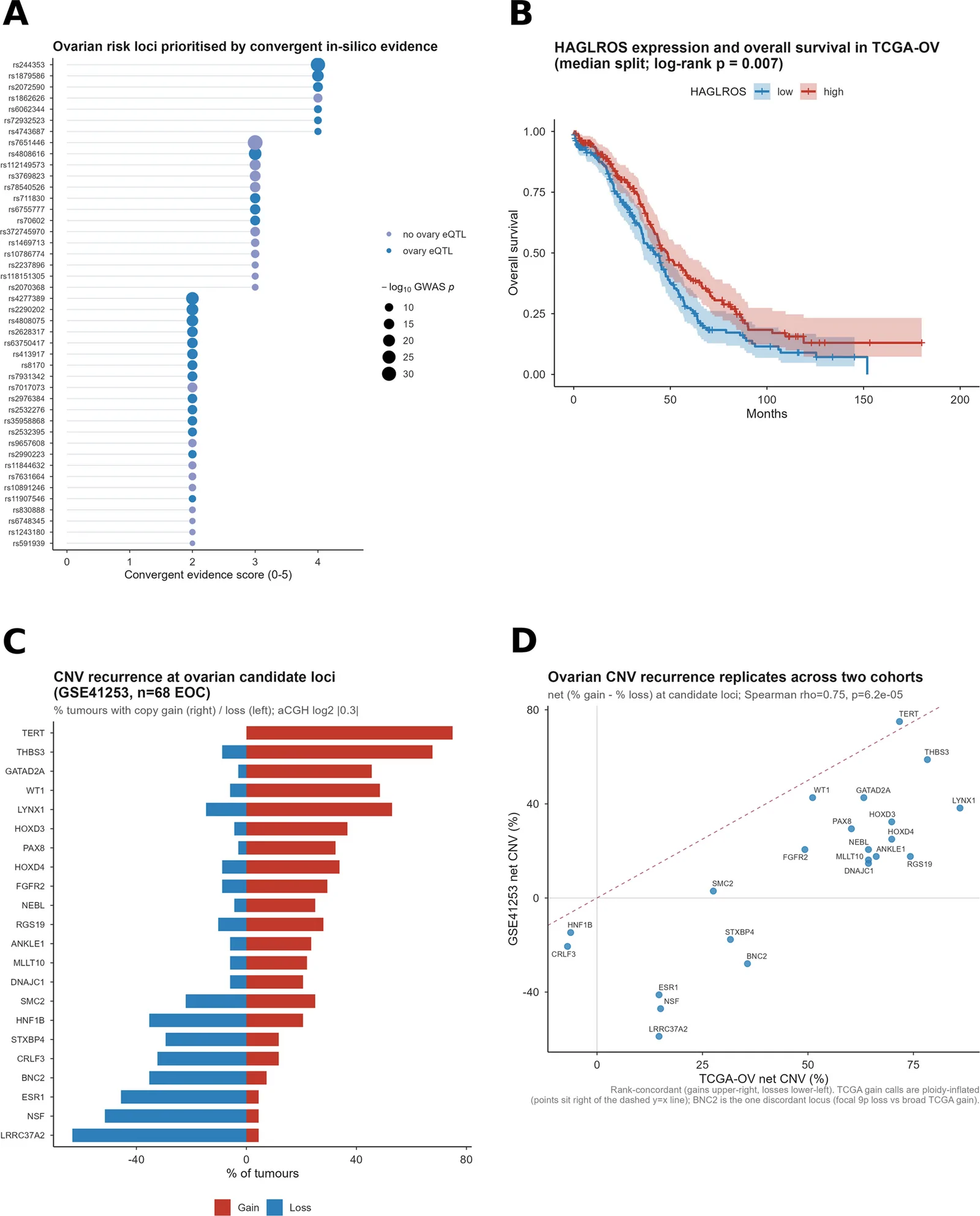
Ovarian cancer risk loci and copy-number recurrence. A Ovarian GWAS leads prioritised by convergent annotation evidence (score ≥ 2); filled symbols mark the robust core from the sensitivity analysis. B HAGLROS expression and overall survival in TCGA-OV (nominal log-rank *p* = 0.007; FDR = 0.30). C Copy-number recurrence at candidate genes in GSE41253. D Cross-cohort agreement of net copy-number alteration at candidate genes; exploratory, because agreement is similar for chromosome-arm-matched control genes (Supplementary Figure S9)

Given these limitations, we framed the ovarian analysis as a hypothesis-generating convergent-evidence prioritisation. Among 176 genome-wide-significant ovarian GWAS leads (p < 5 × 10⁻8), 26 overlapped Ensembl regulatory features, 41 were GTEx ovary eQTLs, and 86 disrupted a TF motif. Convergent-evidence scoring identified 44 candidates at score ≥ 2 and 21 at score ≥ 3 (Fig. 3A; scores of all 176 leads in Supplementary Table S6). After distance-based clumping (500-kb window), the 173 mappable leads collapsed to 92 independent loci (47% redundancy) and the 44 candidates to 31 independent loci (Table 1). Because the prostate benchmark showed these annotations to be weak predictors, we tested how strongly the candidate set depends on scoring choices (Supplementary Table S7, Supplementary Figure S8). Leave-one-feature-out scoring retained 26–40 candidates (Jaccard overlap with the main set 0.59–0.91), alternative weightings retained 26–35 (Jaccard 0.59–0.80), and 2,000 random weightings gave a median Jaccard overlap of 0.83. Candidate membership was therefore sensitive to individual features, particularly strong motif disruption, but all 21 SNPs with score ≥ 3 (17 loci) were selected in at least 80% of deterministic schemes and random weightings; we regard only this robust core as prioritised. Weighting only the two features that carried signal in the prostate benchmark (motif and eQTL) produced a smaller, partly different set (20 SNPs, 12 loci, including 6 of the 21 core SNPs). An experimental criterion could not be applied systematically: only 11 of the 176 leads were tested in the independent ovarian MPRA (GSE183301), of which one (rs2976384, score 2) showed allele-specific activity. Leading candidates included rs4808616 (ANKLE1), rs2072590/rs6755777/rs711830 (HOXD3/HAGLROS), rs244353 (STXBP4), rs4743687 (SMC2), and rs1862626 (disrupting an ESR1 motif). These candidates were strongly enriched in ovary and fallopian-tube regulatory chromatin (Fisher OR = 7.9, p = 1.3 × 10⁻5, FDR = 5 × 10⁻5; robust core OR = 16.9), although this enrichment is partly non-independent of the Ensembl regulatory features used for scoring.

**Table 1:** Ovarian candidate loci prioritised by convergent annotation evidence (score ≥ 3); all 21 SNPs form the robust core of the sensitivity analysis. Score is the sum of five binary features (0–5). NES, normalised effect size of the GTEx v10 ovary eQTL; “–”, no eQTL gene, no mapped gene or no strong motif disruption. These candidates are hypothesis-generating

SNP	GWAS p	Score (0–5)	Target gene(s)	Top disrupted TF motif	Ovary eQTL gene	eQTL NES	Promoter/enhancer	Fallopian-tube ATAC	Regulatory biotype
rs244353	1.0e − 31	4	STXBP4	HNF4A	STXBP4	−0.37	Yes	No	enhancer
rs1879586	2.0e − 19	4	DND1P1;ENSG…	REST	LINC02210	1.18	Yes	Yes	promoter
rs2072590	5.0e − 14	4	HAGLR;HAGLROS;HOXD3;HOXD4	NR1I2	HAGLROS	−0.73	Yes	Yes	enhancer
rs1862626	4.0e − 12	4	Intergenic	ESR1	–	–	Yes	No	enhancer
rs6062344	5.0e − 10	4	RGS19	RUNX3	RGS19	0.20	Yes	No	enhancer
rs72932523	1.0e − 09	4	SMIM38	YY1	SMIM38	0.95	Yes	No	enhancer
rs4743687	5.0e − 09	4	SMC2	STAT2	SMC2	−0.29	Yes	Yes	enhancer
rs7651446	2.0e − 34	3	–	HAND1	–	–	Yes	No	enhancer
rs4808616	2.0e − 23	3	ANKLE1	TEAD4	ANKLE1	0.36	Yes	Yes	promoter
rs112149573	2.0e − 17	3	–	EGR4	–	–	Yes	No	promoter
rs3769823	1.0e − 16	3	–	IRF1	–	–	Yes	Yes	enhancer
rs78540526	1.0e − 15	3	–	ZNF350	–	–	Yes	No	enhancer
rs6755777	3.0e − 15	3	HAGLROS;HOXD3;HOXD4	–	HAGLROS	−0.71	Yes	Yes	promoter
rs711830	3.0e − 15	3	HAGLR;HAGLROS;HOXD3;HOXD4	–	HAGLROS	−0.74	Yes	No	enhancer
rs70602	6.0e − 14	3	ARL17A;ENSG…	–	ENSG00000262539	−1.20	Yes	No	enhancer
rs372745970	2.0e − 13	3	–	IRF3	–	–	Yes	Yes	enhancer
rs10786774	1.0e − 10	3	–	NRF1	–	–	Yes	No	enhancer
rs1469713	1.0e − 10	3	GATAD2A	ZBTB18	–	–	Yes	No	enhancer
rs2237896	5.0e − 09	3	–	TFAP2B	–	–	Yes	Yes	enhancer
rs118151305	6.0e − 09	3	–	PAX6	–	–	Yes	Yes	enhancer
rs2070368	1.0e − 08	3	–	PBX2	–	–	Yes	No	enhancer

### Candidate genes lie in recurrently altered regions, but not more than matched control genes

Testing candidate target-gene expression against overall survival in TCGA-OV, no gene survived multiple-testing correction (all log-rank FDR > 0.30; all Cox FDR > 0.75), though HAGLROS gave the strongest nominal signal (log-rank p = 0.007, FDR = 0.30; Cox HR = 0.93, p = 0.06), alongside NEBL (log-rank p = 0.10, Cox p = 0.02, HR = 1.18, Cox FDR = 0.75) (Fig. 3B); these nominal associations are not interpreted further. Candidate loci showed recurrent copy-number alterations across two independent cohorts (TCGA-OV, n = 272; GSE41253, n = 68) (Fig. 3C), and net alteration frequencies at the 22 candidate genes measured in both cohorts were correlated (Spearman ρ = 0.75, bootstrap 95% CI 0.46–0.88, permutation p = 2 × 10⁻4) (Fig. 3D). The correlation was not driven by extreme genes (jackknife range 0.72–0.80; ρ = 0.70 after removing BNC2 and the four most altered genes), and MECOM, annotated as EVI1/MDS1 on the aCGH platform, was not part of the 22-gene comparison. However, the two cohorts agreed to a similar degree across all 14,045 genes measured in both (ρ = 0.66), and chromosome-arm-matched random sets of 22 genes gave a median ρ of 0.64 (95% range 0.30–0.86; empirical p = 0.21 for the candidates); candidate genes were only marginally more frequently altered than matched controls (mean absolute net alteration 31% vs 25%, empirical p = 0.06; Supplementary Table S9, Supplementary Figure S9). The cross-cohort agreement therefore reflects the shared copy-number landscape of high-grade serous ovarian cancer rather than a property specific to the candidates, and we report it as exploratory. Recurrent gains occurred at lineage/oncogenic candidates including MECOM (89% gain, 50% amplified in TCGA), TERT, PAX8, WT1, the HOXD cluster, and ANKLE1, while recurrent losses mapped to tumour suppressors such as BNC2, ESR1, and HNF1B.

### Cross-cancer synthesis generates a hypothesis of shared IRF/STAT motif disruption

**Fig. 4:**
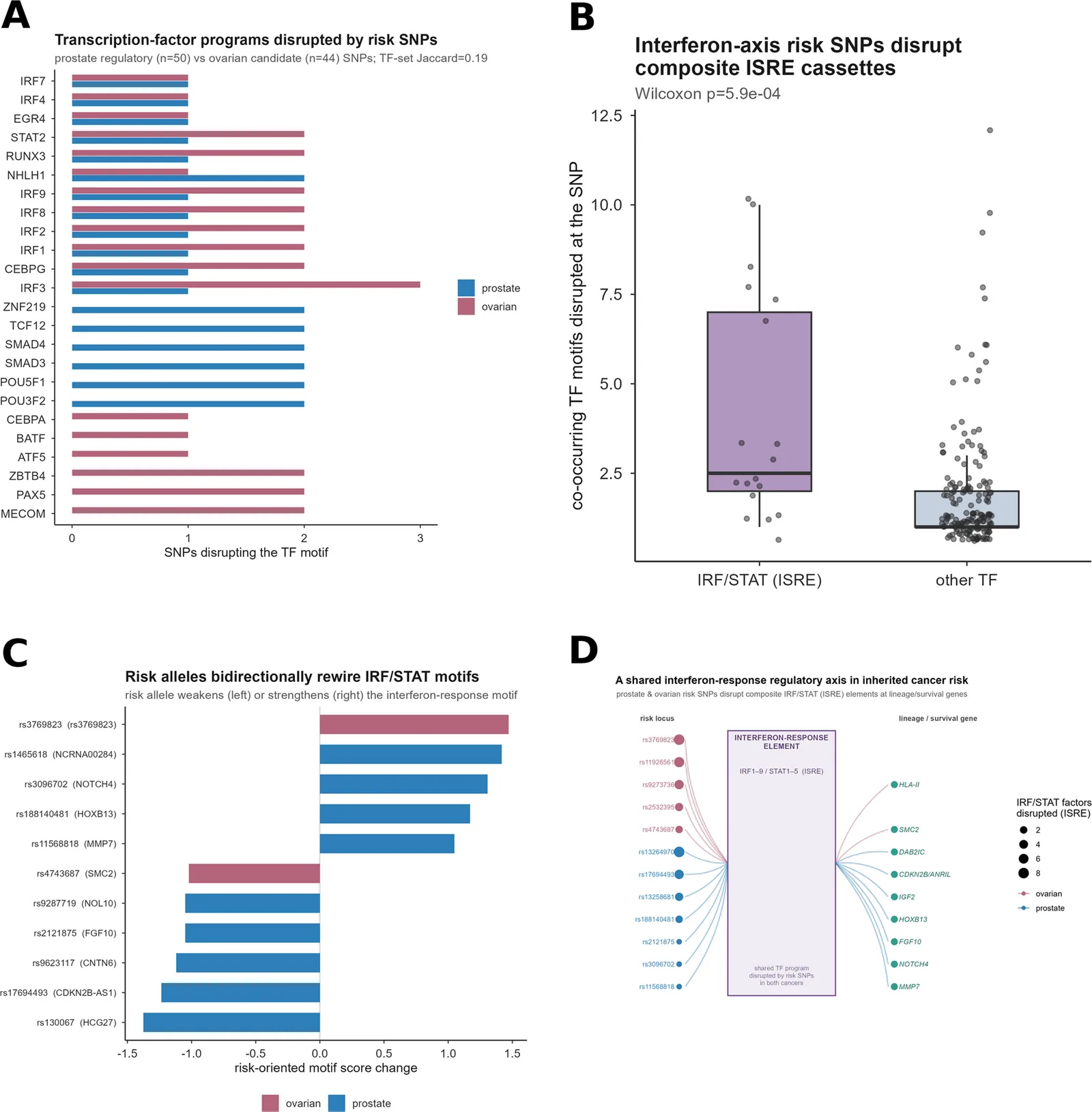
Transcription-factor motifs disrupted by prostate and ovarian risk SNPs and the hypothesised IRF/STAT motif signature. A TF motifs disrupted (motifbreakR, strong effects) by prostate regulatory SNPs and ovarian candidate SNPs, grouped as shared or cancer-specific. B Number of co-occurring motifs disrupted at SNPs that disrupt an IRF/STAT motif versus other motif-disrupting SNPs. C Risk-allele-oriented change in predicted IRF/STAT motif scores. D Schematic of the hypothesised IRF/STAT motif signature; links to genes are eQTL/GWAS nominations, and the hypothesis was not supported by the primary ovarian MPRA (Fig. 7C). FDR values are Benjamini–Hochberg adjusted (Supplementary Table S10)

Intersecting disrupted TF motifs between prostate regulatory SNPs and ovarian candidates revealed modest overall overlap (Jaccard index 0.19) (Fig. 4A), but the shared fraction was dominated by the interferon-regulatory factor (IRF) and STAT families: IRF1/2/3/4/7/8/9 and STAT2 motifs were disrupted in both malignancies (8 of 20 shared TFs vs 0 of 88 TFs disrupted in only one cancer; Fisher FDR = 3 × 10⁻6). This over-representation should be interpreted cautiously, because IRF position weight matrices are highly similar and a single SNP typically disrupts several of them. Eighteen risk SNPs (12 prostate, 6 ovarian) disrupted these motifs, frequently hitting multi-factor ISRE cassettes (up to IRF1–9 + STAT2 at a single SNP; more co-occurring motifs than at other motif-disrupting SNPs, Wilcoxon p = 6 × 10⁻4, FDR = 0.002) (Fig. 4B). Risk alleles had no consistent direction of effect on these predicted elements—weakening the ISRE at 6 loci and strengthening it at 5 orientable loci (Wilcoxon p = 0.69) (Fig. 4C)—and the corresponding SNPs are nominated by eQTL or GWAS mapping to regulate lineage and survival genes including HOXB13, IGF2, DAB2IC, CDKN2B/ANRIL, FGF10, NOTCH4, MMP7, SMC2, and HLA-II (Fig. 4D). Together these observations define a motif-level hypothesis of shared IRF/STAT motif disruption, which we tested experimentally below.

### Pleiotropic loci and formal colocalisation at flagship loci

**Fig. 5:**
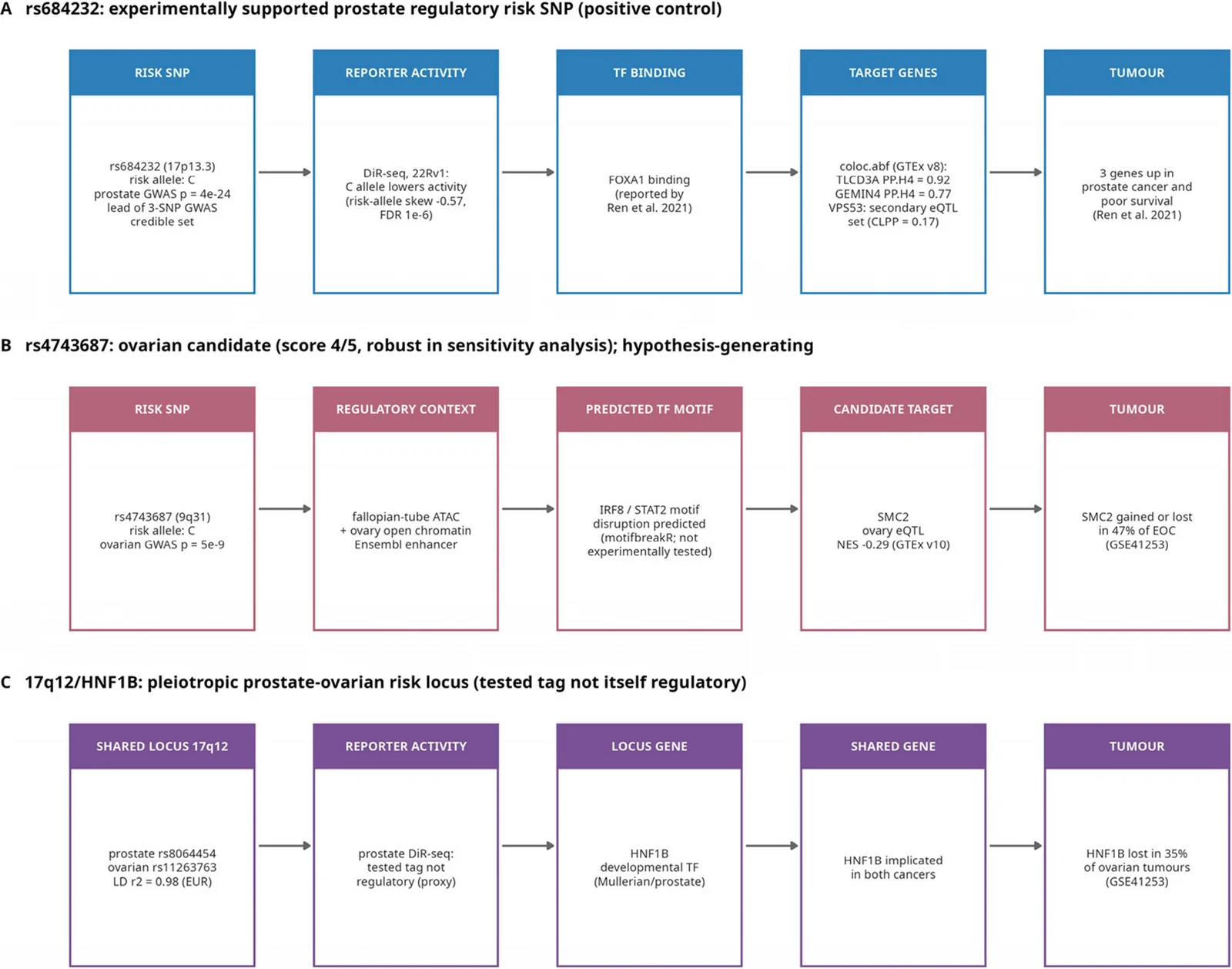
Flagship risk loci traced from allele to regulatory element, candidate target gene and tumour. **A** rs684232 at 17p13.3, an experimentally supported prostate regulatory SNP; target genes are supported by formal colocalisation (TLCD3A, GEMIN4) or by a secondary VPS53 eQTL credible set. **B** rs4743687, a robust ovarian candidate (score 4/5) with a predicted, untested IRF8/STAT2 motif disruption; the link to SMC2 is an eQTL nomination and is hypothesis-generating. C The pleiotropic 17q12/HNF1B locus, where the tested prostate tag SNP is not itself regulatory

**Fig. 6:**
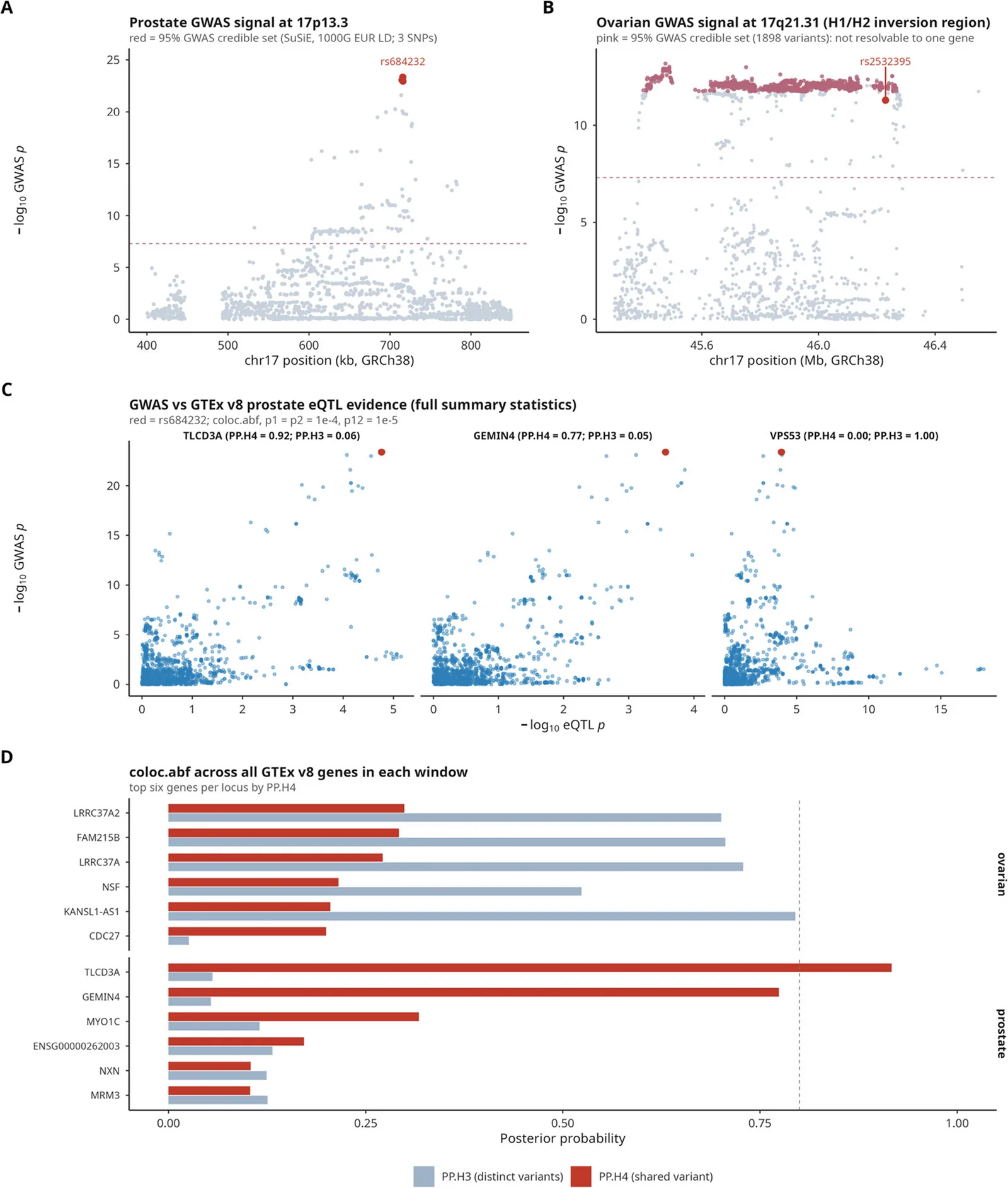
Formal colocalisation at the flagship loci using full summary statistics. **A** Prostate cancer GWAS signal at 17p13.3; red points form the 95% GWAS credible set (SuSiE-RSS, 1000 Genomes EUR LD). **B** Ovarian cancer GWAS signal at 17q21.31; the 95% credible set (pink) spans 1,898 variants within the H1/H2 inversion region and cannot be resolved to a single gene. **C** GWAS versus GTEx v8 prostate eQTL evidence for TLCD3A, GEMIN4 and VPS53, with coloc.abf posteriors (PP.H4, shared causal variant; PP.H3, distinct causal variants). **D** coloc.abf posteriors for the six genes with the highest PP.H4 in each window; dashed line, PP.H4 = 0.8

The two cancers also share specific risk loci. Three SNPs tested in the prostate MPRA (rs7725218, rs7679673, rs7931342) are also ovarian GWAS hits but were non-regulatory in the prostate assay, likely tagging distinct causal variants. We identified 10 shared loci in linkage disequilibrium (r2 ≥ 0.2), 7 in strong LD (r2 ≥ 0.8), including TERT (5p15), 8q24/MYC, HNF1B (17q12, r2 = 0.98), 11q13, JAZF1 (7p15), and 17q24 (Supplementary Table S2, Supplementary Figure S7A); shared target genes included HNF1B, RAD51B, and SMIM38 (Supplementary Figure S7B). We then tested formally whether disease and expression signals share causal variants at the two flagship loci, using full GWAS and GTEx v8 summary statistics (Figs. 5 and 6, Supplementary Table S8). At 17p13.3, the prostate GWAS signal fine-mapped to a three-variant 95% credible set led by rs684232 (posterior inclusion probability 0.48; GWAS p = 4.3 × 10⁻24). coloc.abf supported a shared causal variant for prostate cancer risk and TLCD3A (FAM57A) expression (PP.H4 = 0.92) and, more moderately, GEMIN4 expression (PP.H4 = 0.77); PP.H4 for TLCD3A remained above 0.8 at p12 = 5 × 10⁻6 but fell to 0.53 at p12 = 1 × 10⁻6. For VPS53, the primary prostate eQTL signal (lead rs4968120; r2 = 0.01 with rs684232) was distinct from the GWAS signal (PP.H3 = 1.00 and PP.H4 ≈ 0 in both coloc.abf and coloc-SuSiE). However, GTEx v10 fine-mapping resolves a second, independent VPS53 eQTL credible set that contains rs684232 (posterior inclusion probability 0.30), and this set overlapped the GWAS credible set (CLPP = 0.17). The absence of colocalisation for VPS53 under a single-causal-variant model therefore does not contradict previous reports that rs684232 and the 17p13.3 locus regulate VPS53 together with TLCD3A and GEMIN4 [8, 25, 26]; rather, the dominant VPS53 eQTL is driven by a different variant. In coloc-SuSiE, no credible set was detected for the weaker TLCD3A and GEMIN4 eQTLs (minimum eQTL p = 7 × 10⁻6 and 1 × 10⁻4), so multi-variant colocalisation could not be estimated for these genes. At 17q21.31, the ovarian GWAS signal lies within the H1/H2 inversion haplotype: its 95% credible set contained 1898 variants and overlapped the eQTL credible sets of 14 genes, none of the 55 genes tested in GTEx v8 ovary reached PP.H4 above 0.30 (highest, LRRC37A2), and LINC02210 is not included in the GTEx v8 full-statistics release. The colocalisation with LINC02210 reported previously was based on a regional enrichment test, which measures regional overlap rather than a shared causal variant; we therefore no longer claim colocalisation at this locus.

### Independent MPRA in primary epithelial cells supports one ovarian candidate but not the interferon hypothesis

**Fig. 7:**
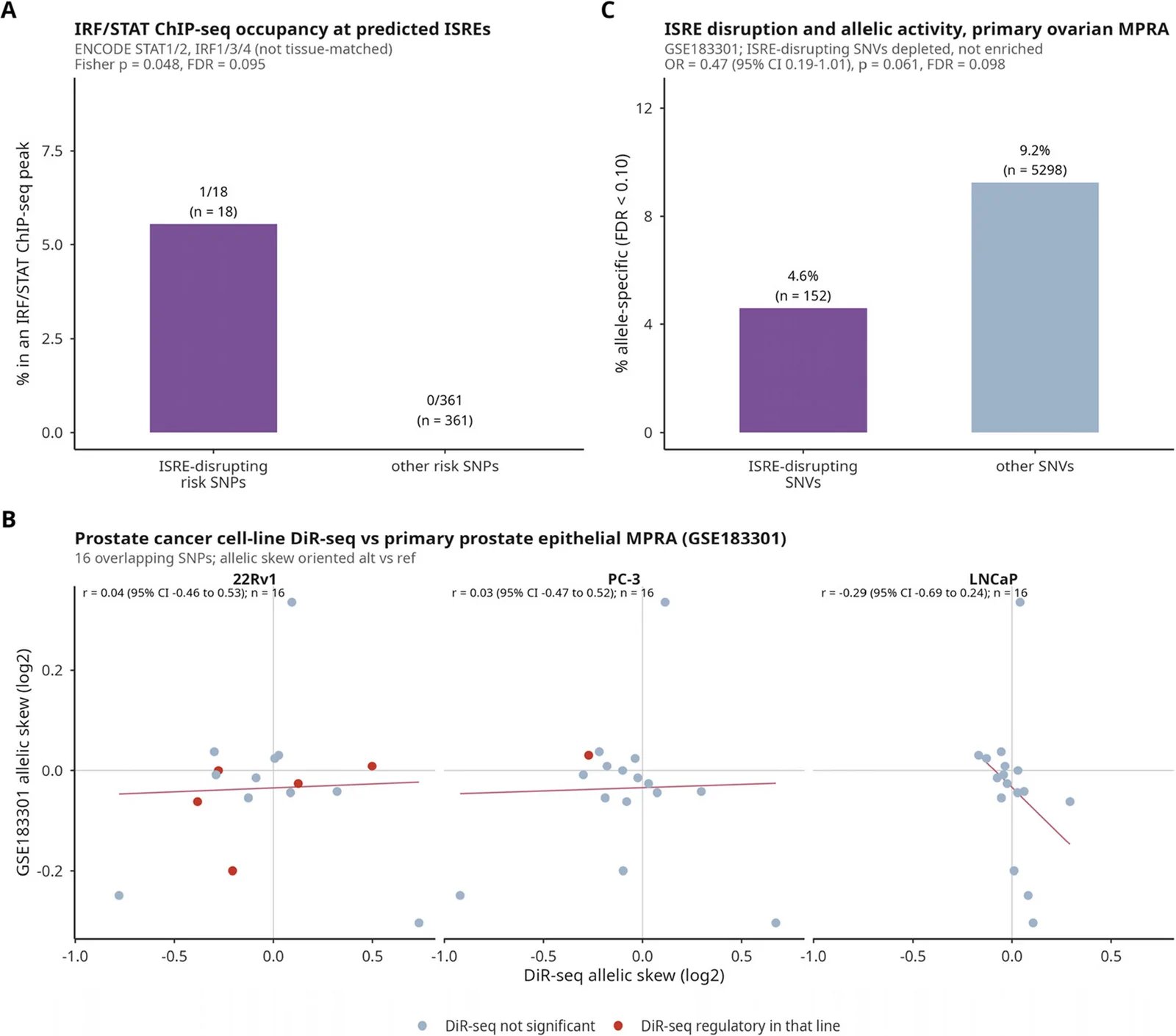
Experimental tests of the IRF/STAT hypothesis and cross-platform replication. A Overlap of ISRE-disrupting risk SNPs with ENCODE IRF/STAT ChIP-seq peaks (not tissue-matched). B Allelic skew in DiR-seq (22Rv1, PC-3 and LNCaP prostate cancer cells) versus the independent GSE183301 MPRA in primary prostate epithelial cells for the 16 shared SNPs, by cell line. C Fraction of allele-specific SNVs (FDR < 0.10) among ISRE-disrupting and other SNVs in the GSE183301 MPRA in primary ovarian epithelial cells; ISRE-disrupting SNVs are depleted, not enriched

Probing physical occupancy of the predicted ISRE sites gave weak support: only 1 of the 18 ISRE-disrupting SNPs fell within public ENCODE STAT1/STAT2/IRF1/3/4 ChIP-seq peaks, compared with 0 of 361 other risk SNPs (Fisher p = 0.048, FDR = 0.095) (Fig. 7A). Cross-platform reproducibility was also low. The independent MPRA (GSE183301) [14] was performed in primary prostate and ovarian epithelial cells rather than cancer cell lines, and 16 SNPs overlapped the DiR-seq set. Allelic skew in primary prostate epithelial cells showed no concordance with DiR-seq in 22Rv1 (Pearson r = 0.04, 95% CI − 0.46 to 0.53), PC-3 (r = 0.03, − 0.47 to 0.52) or LNCaP (r = − 0.29, − 0.69 to 0.24), and effect directions agreed for only 10, 7 and 6 of the 16 SNPs, respectively (Fig. 7B; per-SNP values in Supplementary Table S4). Among the five SNPs regulatory in 22Rv1, the correlation was r = 0.43 (p = 0.47). Only one of the 16 SNPs (rs11704416) was allele-specific in GSE183301, and it was not regulatory in DiR-seq, so the comparison is dominated by small effects close to zero in both assays. To validate the ovarian candidates and test the interferon hypothesis, we re-analysed GSE183301 in primary ovarian epithelial cells, yielding 497 allele-specific SNVs (FDR < 0.10; 310 at 0.05) and confirming allele-specific activity at the prioritised candidate rs2976384 (convergent score 2; allelic skew = − 0.67, FDR = 3 × 10⁻5). The interferon hypothesis, however, was not supported: ISRE-disrupting SNVs were depleted rather than enriched for activity (7 of 152, 4.6% active) versus non-disrupting SNVs (490 of 5298, 9.2%; Fisher OR = 0.47, 95% CI 0.19–1.01, nominal *p* = 0.06, FDR = 0.10) (Fig. 7C).

## Discussion

We showed that 50 prostate cancer risk SNPs exert robust, allele-specific regulatory activity in at least one of three prostate cancer cell lines (30 at FDR < 0.05), establishing an FDR-controlled catalogue. Benchmarking these readouts against standard in-silico annotations revealed that computational predictions—even with cell-type-matched epigenomics—perform only marginally better than chance. On this basis, the ovarian analysis was framed as hypothesis-generating: convergent annotation prioritised 31 candidate loci, of which a core of 17 loci was robust to the choice of features, weights and thresholds. The cross-cancer synthesis showed direct pleiotropy at several loci and an over-representation of IRF/STAT motifs among motifs disrupted in both cancers, but this motif-level hypothesis was not supported experimentally. Building on foundational GWAS of these cancers [17, 18], the poor predictive performance of annotations underscores the necessity of empirical validation, echoing recent MPRA studies of context-specific enhancer activity [27–29]; our audited catalogue provides a reliable foundation for dissecting the non-coding architecture of these hormone-dependent malignancies.

We observed substantial pleiotropy connecting prostate and ovarian risk networks, particularly at HNF1B and TERT, consistent with the known dual role of HNF1B across both malignancies [2]. Epigenetic silencing and allele-specific expression of HNF1B modulate epithelial-to-mesenchymal transition as a subtype-specific ovarian susceptibility mechanism [30–32], while TERT is independently associated with telomere length and risk of both cancers, implying a shared vulnerability in chromosomal maintenance [33–35]. Variants at such pleiotropic loci are frequently associated with the expression of nearby genes [36–38], but regional overlap of GWAS and eQTL signals does not establish a shared causal variant. With formal colocalisation, we found support for a shared variant at 17p13.3 for TLCD3A and GEMIN4 but not for the primary VPS53 eQTL, and the 17q21.31 ovarian signal could not be resolved because of extensive LD across the inversion haplotype.

Among the prioritised ovarian candidates, we found recurrent copy-number alterations at MECOM, BNC2, and the 17q21.31 region (LRRC37A2, NSF). MECOM is frequently amplified in aggressive epithelial ovarian cancers, with overexpression linked to immunosuppression and poor outcomes [39, 40]; 9p22 variation encompassing BNC2 is associated with sonographic ovarian abnormalities and altered developmental transcription [41–43]; and structural changes at 17q21.31 disrupt the local regulatory network [44–46]. However, candidate genes were not more concordantly altered across cohorts than chromosome-arm-matched control genes. These observations show that some candidate genes lie in recurrently altered regions of the high-grade serous genome, not that germline risk variants prime these regions for somatic disruption; any link between inherited risk and acquired copy-number change remains a hypothesis.

At the flagship prostate locus, rs684232 was both experimentally regulatory and the lead of a three-variant GWAS credible set; formal colocalisation supported TLCD3A and GEMIN4 as target genes, and a secondary VPS53 eQTL signal containing rs684232 was consistent with earlier work. VPS53 is an established target of prostate risk loci, with multi-omic and transcriptomic analyses confirming allele-specific binding and altered expression in prostate cells [25, 47–49], and rs684232 was originally shown to alter FOXA1 binding and the expression of VPS53, FAM57A (TLCD3A) and GEMIN4 [8]. Regulatory variants near MMP7 disrupt transcription-factor binding within active enhancers to drive context-specific expression [26, 50, 51]. Recovering these established targets with formal colocalisation and an FDR-controlled catalogue indicates that the pipeline captures genuine biological signal, and also shows that the dominant eQTL at a locus need not share the risk variant.

The shared transcription-factor signature centred on disruption of interferon-regulatory factor (IRF) and STAT motifs. Although the IRF family is widely implicated in cancer survival and immunomodulation [13, 52], our experimental test did not confirm that ISRE-disrupting variants are more active; in primary ovarian epithelial cells they were, if anything, depleted for allele-specific activity. The over-representation of IRF/STAT motifs may partly reflect the redundancy of IRF position weight matrices, so that a few SNPs contribute many shared motifs. The low concordance between our DiR-seq re-analysis and the independent MPRA is likely to reflect differences in cellular context (prostate cancer cell lines versus primary epithelial cells) and in assay design (55-bp inserts and one barcode per allele in DiR-seq [8] versus ten barcodes per allele in GSE183301 [14]), together with the small number of overlapping SNPs with appreciable effects in either assay; such platform- and context-specific effects are well documented for MPRAs [5, 27–29]. IRF and STAT factors have context-dependent roles in tumour suppression and immune evasion that episomal reporters may not capture [13, 52]. This null result reinforces the study’s central caution: motif-level predictions are often uncoupled from measured function and require empirical validation.

Several limitations apply. The ovarian fine-mapping relies on convergent annotation evidence and remains hypothesis-generating; candidate membership outside the robust core depends on scoring choices, and only 11 ovarian leads could be assessed experimentally. Colocalisation at 17q21.31 is limited by the extended LD of the inversion haplotype, coloc-SuSiE could not be estimated for weak eQTLs, and the LD reference (1000 Genomes EUR, n = 503) is much smaller than the GWAS samples. The cross-cohort copy-number agreement is not specific to candidate genes, and no survival association survived FDR correction. The lack of endogenous chromatin context in episomal MPRAs may also explain the failure to validate the interferon hypothesis [27, 28]; chromatin state and three-dimensional organisation shape regulatory activity and enhancer–gene contacts [10–12], which neither episomal reporters nor sequence-based annotations capture. Future work should prioritise tissue-matched allele-specific binding assays, CRISPR-based endogenous enhancer perturbations, and in-vivo validation to test whether IRF/STAT motif disruption has any functional role, and Hi-C-based spatial proximity, as recently applied to splicing QTLs in cancer tissues [12], could help link the robust ovarian candidates to target genes beyond eQTL nomination. Integrating such models with our audited catalogue will be essential for translating non-coding associations into clinical insight.

## Conclusion

This study provides an FDR-controlled catalogue of prostate cancer regulatory SNPs and shows the stark limits of in-silico annotations as proxies for measured enhancer activity. Convergent annotation evidence generated a robust set of hypothesis-generating ovarian candidates, one of which showed allele-specific activity in an independent assay, and formal colocalisation supported TLCD3A and GEMIN4 as targets of the rs684232 prostate risk locus. Cross-cancer synthesis identified shared pleiotropic loci and a predicted IRF/STAT motif signature, but this signature was not supported experimentally. These findings emphasise the need for context-specific experimental validation in functional fine-mapping of complex disease loci.

## Supplementary Information

Supplementary Material 1.

Supplementary Material 2.

Supplementary Material 3.

## Data Availability

No datasets were generated or analysed during the current study.
