# Supplementary material for: Experimental benchmarking of in silico annotations at prostate cancer risk loci and hypothesis-generating cross-cancer prioritisation of ovarian risk variants
Source: Genomics Inform. 2026 Oct 28;24:24. doi: 10.1186/s44342-026-00087-2 (PMC13718048; doi:10.1186/s44342-026-00087-2)
Supplement: Supplementary file 1 [file 44342_2026_87_MOESM1_ESM.docx]

# Supplementary Figures

**Experimental Benchmarking of In-Silico Annotations at Prostate Cancer Risk Loci and Hypothesis-Generating Cross-Cancer Prioritisation of Ovarian Risk Variants**

**Supplementary Figure S1. Library correlation matrix demonstrating high replicate consistency across DiR-seq samples.**

**Supplementary Figure S2. DiR-seq library quality control and sanity checks. (A) Scatter plot of RNA versus plasmid DNA tag counts in 22Rv1 cells (replicate 1). (B) Total read counts across the 22 retained DiR-seq libraries. (C) Sanity check confirming the recovery of the rs684232 allelic reporter activity.**

**Supplementary Figure S3. Bar chart categorising regulatory SNPs into shared, cell-specific, and shared-discordant classes.**

**Supplementary Figure S4. Regulatory SNPs and reporter activity. (A) Comparison of regulatory SNP counts between the original study and our FDR-controlled re-analysis. (B) Risk-allele effect on reporter activity for 22Rv1 regulatory SNPs, showing bidirectional effects.**

**Supplementary Figure S5. Functional-annotation evidence for the 50 regulatory SNPs: strong TF-motif disruption, Ensembl regulatory feature, promoter/enhancer overlap, GTEx v10 prostate eQTL, and a reported GWAS prostate risk allele (38 of 50 SNPs).**

**Supplementary Figure S6. Prediction of experimental regulatory SNPs from annotations. (A) ROC curves for predicting experimental regulatory SNPs from annotations. (B) Annotation feature importance derived from the random forest classifier. (C) Impact of adding cell-type epigenomics on the prediction of MPRA activity.**

**Supplementary Figure S7. Shared prostate-ovarian risk loci and genes. (A) Shared prostate-ovarian risk loci in linkage disequilibrium. (B) Genes implicated in BOTH prostate and ovarian cancer.**

**Supplementary Figure S8. Sensitivity of the ovarian convergent-evidence prioritisation. (A) Jaccard overlap between the main candidate set (score ≥ 2) and the candidate sets obtained with alternative thresholds, leave-one-feature-out scoring, alternative weightings and definitions, and 2,000 random Dirichlet weightings; labels give the number of candidate SNPs and independent loci. (B) Fraction of the 12 deterministic scoring schemes that select each main candidate; dashed line, 80% robustness threshold.**

**Supplementary Figure S9. Robustness of the cross-cohort copy-number agreement at candidate genes. (A) Net copy-number alteration (% gain − % loss) at the 22 candidate genes in TCGA-OV and GSE41253, with the bootstrap 95% confidence interval of Spearman ρ. (B) Cross-cohort Spearman ρ for 10,000 random sets of 22 genes matched to the candidates by chromosome arm; red line, candidate genes (empirical p = 0.21).**
